# Study on the production of Sophorolipid by *Starmerella bombicola* yeast using fried waste oil fermentation

**DOI:** 10.1042/BSR20230345

**Published:** 2024-01-31

**Authors:** Haifeng Wang, Ruifang Gao, Xin Song, Xiangdong Yuan, Xiuli Chen, Yanling Zhao

**Affiliations:** 1 Department of Food and Drug, Baotou Light Industry Vocational Technical College, Baotou, Inner Mongolia, China; 2 Modern Agriculture and Animal Husbandry Development Center, Bureau of Agriculture and Animal, Husbandry of Bayannur City, Bayannur, Inner Mongolia, China; 3 Institute of Microbial Technology, Shandong University, Qingdao, Shandong, China; 4 School of Biological Science and Technology, Baotou Teachers’ College, Baotou, Inner Mongolia, China

**Keywords:** Fried waste oil, Sophorolipid, Starmerella bombicola

## Abstract

Sophorolipids (SLs) are surface active compounds that have excellent surface-lowering properties. SLs were produced by *Starmerella bombicola* (CGMCC1576) yeast with sunflower seed oil, fried waste oil, cooked tung oil and raw tung oil used as hydrophobic carbon sources. The results showed that the strain could use sunflower seed oil and fried waste oil as hydrophobic carbon sources to produce SLs, and the yields were 44.52 and 39.09 gl^−1^. It could not be used as cooked tung oil and raw tung oil. The analysis by high-performance liquid chromatography/high resolution mass spectrometry (HPLC-MS/MS) showed that the main composition and structure of SLs produced by fermentation using fried waste oil were similar to that of sunflower seed oil as hydrophobic carbon source. The yield of SLs was the highest when the fried waste oil was used as hydrophobic carbon source, glucose (8%), waste oil (6%) and yeast (0.3%). When fried waste oil was used as a hydrophobic carbon source in a parallel 4-strand fermentation tank (FT), the combination with the largest yield and the most cost saving was that 3% of fried waste oil was added into the initial medium, and another 3% was again added after 72 h of fermentation. The total yield of SLs was 121.28 gl^−1^, and the yield of lactone SLs was 48.07 gl^−1^.

## Introduction

Biosurfactant was given more attention by people in recent years than others [1–4]. Biosurfactants have many advantages, including surface activity, foaming ability, thermal stability, emulsifying ability, better environmental compatibility and greater specificity [5–7]. They are nontoxic [8,9], biodegradable and prevent the secondary pollution of the environment. They are generally nonsensitive and digestible, and due to these characteristics, the replacement of chemical synthetic surfactants will become the mainstream trend and have a good development prospect [11–18]. Production materials are less expensive and easy to get, which can be obtained from industrial and agricultural wastes biologically, reproducibly and cyclically [10].

There are various kinds of biological surfactants produced by microorganisms with different structures, which can be roughly divided into five categories according to their sources and structural characteristics. These include glycolipids, liposeptides and lipoproteins, phospholipids and fatty acids, polymeric surfactants and particulate surfactants [10,19].

SLs were first discovered in 1954 [20]. They are kinds of biosurfactant produced mainly by nonpathogenic yeast, which are divided into acid type and lactone type [21]. Due to above advantages, it has a good application prospect in many fields such as food, medicine, petroleum, chemical industry and environmental remediation [1,5,11,22–28]. At present, SLs are mainly produced by microbial fermentation, and generally consist of hydrophilic and hydrophobic carbon sources. When only hydrophilic or hydrophobic carbon sources are present, SLs can be synthesized, but the yield is generally low. As a surfactant, SLs are not used in large scale, and the main reason is that the production cost is much higher than that of chemical surfactant.

In production, the cost of fermentation raw materials alone can be up to more than 50%, of which carbon sources account for the main part [29,30]. Therefore, it is one of the most effective ways to reduce production cost to choose less expensive substrates to replace the carbon source, and at the same time to improve yield and reduce cost through optimization of fermentation process.

In the present study, the capacity of producing SLs and its structure under different culture conditions were selected to investigate under different hydrophobic carbon sources. Orthogonal design method was used to optimize the composition of medium and culture conditions, and the culture was expanded in fermentation tanks to provide reference for future production.

## Materials and methods

### Strain

The producing strain of SLs was *Starmerella bombicola* (CGMCC1576) was provided by the Institute of Microbial Technology, Shandong University.

### Instruments and reagents

#### Experimental instruments

The following instruments were used in the study: LD2X-40B1 type vertical automatic pressure steam sterilizer (Shanghai ShenAn Medical Instrument Factory), super clean workbench (China Suzhou Purification Equipment Factory), HDK-8D type thermostatic tank (Shanghai SenXin Experimental Instrument Co., Ltd), thermostatic incubator DHP-500 (Tianjin Zhonghuan Experimental Electric Furnace Co., Ltd.), biosensor SBA-40C (Shandong Academy of Sciences), RE5203 type rotary evaporation plant (Shanghai YaRong Biochemical Instrument Factory), SpectraMax M5 multifunctional enzyme mark (MolecularDevices, U.S.A.), Agilent 1100 series HPLC analysis system (Shimadzu, Japan), Venusil MP C18 analysis column (4.6 µm × 250 mm × 4.6 mm, Agela Technologies Inc., U.S.A.), API 4000 mass spectrometer (Applied Biosystems, U.S.A.) and Quadruplex parallel fermentation tank Multifors2/1.4l (INFORS, Switzerland).

#### Reagent

Acetonitrile, chromatographic pure was purchased from BaiWeiLing Technology Co., Ltd. Anthrone was purchased from Sigma Biological Technology Co., Ltd. (U.S.A.). Glucose, ethanol, ethyl acetate and so on are analytically pure and were purchased from Group Pharmaceutical Co., Ltd. Sunflower seed oil, raw tung oil and cooked tung oil were purchased from the local market. The fried waste oil is composed of sunflower oil collected after continuous frying, provided by the canteen.

### Methods

#### Culture medium and culture method

##### Seed culture

YEPD seed medium (w/v): glucose 2.0%, protein 2.0%, yeast 1.0%, AGAR 2.0% (not added during liquid seed culture). Approximately 50 ml of seed medium was put into 300 ml triangular bottles, and after sterilization, the inoculation quantity was 2% (v/v), which was cultured at 200 rpm at 30°C for 16 h.

##### Fermentation culture

Using glucose as hydrophilic carbon source, sunflower seed oil, fried waste oil, cooked tung oil and raw tung oil were used as hydrophobic carbon source for fermentation. The medium was configured as (w/v): glucose, 8%; yeast, 0.3%; KH_2_PO_4_, 0.1%; Na_2_HPO_4_·12H_2_O, 0.1%; MgSO_4_·7H_2_O, 0.1%; hydrophobic carbon source, 6% (v/v). The prepared 50 ml of fermentation medium was put into 300 ml triangular bottles, sterilized, inoculated at 2% (v/v) into the seed solution and cultured at 200 rpm at 30°C for 7 days. The fermentation broth biomass, glucose residue, lactone type and total SLs yield were measured.

#### Optimization of fermentation medium

Based on the choice of fried waste oil as the hydrophobic carbon source, glucose, fried waste oil and yeast powder are the primary components in the fermentation process of SLs. They were designed into different concentrations (glucose: 6,8,10%; fried waste oil: 6,8,10%; yeast powder: 0.2,0.3,0.4 /%), and single factor (not listed in this study) and orthogonal test.

#### Test of fried waste oil by using four-strand parallel fermentation tank

Based on the above test results, glucose was used as the hydrophilic carbon source and fried waste oil as the hydrophobic carbon source to study the effects of different flow dosages of fried waste oil on the effect of fermentation after 3 days. The experimental design was as follows, amount of fried oil was initially 6%. After 3 days, fried oil reenter: 0% for tank 1, 1% for tank 2, 2% for tank 3, and 3% for tank 4. In addition to fried waste oil, the medium was prepared as follows: glucose, 8%; yeast, 0.3%; KH_2_PO_4_, 0.1%; Na_2_H PO_4_·12H_2_O, 0.1%; MgSO_4_·7H_2_O, 0.1%. The experiment was carried out in a four-part parallel fermentation tank, and 1000 ml fermentation medium was loaded into each fermentation tank and inoculated at the inoculation volume of 2% (v/v). The culture was conducted at 25°C and 700 rpm for 7 days. The initial pH of the fermentation liquid was neutral, and when the pH reached 3.5, NaOH was added to maintain the pH 3.5 [31]. Glucose residue, fermentation broth biomass, lactone SLs and total SLs were measured by sampling every 12 h.

#### Detection method

##### Determination of biomass

The solid residue was washed twice with distilled water, and the cell pellet was dried at 50°C to a constant weight. Take 2 ml of fermentation liquid, add eluent of equal volume (V ethanol: V n-butanol: V chloroform = 10:10:1), mix well, centrifuge for 10 min at 8,000 rpm, wash twice with distilled water, and weigh dry weight after drying to constant weight. [32]

##### Determination of glucose concentration

After proper dilution of the fermentation liquid, 25 µl was taken and the residual amount of glucose in the fermentation liquid was determined by using SBA-40C biosensor, repeated three times and averaged.

##### Measurement of the yield of SLs

The yield of locust fat was determined by anthranone–sulfuric acid method [33].

##### Analysis of composition of SLs

Sophorolipid Compositional Analysis Two milliliters of high performance liquid chromatography (HPLC) grade acetonitrile was added to 1 ml cultivation broth and then centrifuged at 10,000 rpm for 10 min. The supernatant was collected after the mixture was dried at 50 °C. The total sophorolipids were obtained and redissolved in HPLC grade acetonitrile. The components of total sophorolipids were analyzed by HPLC with a 4.6-μm column of Venusil MP-C18 (250 mm×4.6 mm, Agela Technologies Inc., USA). Acetonitrile/water was used as the mobile phase by an acetonitrile gradient from 40 to 90 % in 40 min and at a flow rate of 1.0 ml/min. The injection volume was 20 μl and the eluent was monitored with a UV detector at 207 nm. [34] Approximately 1 ml of fermentation liquid was added to 2 ml of chromatographic grade acetonitrile, mixed and centrifuged at 10,000 rpm for 10 min. Supernatant was collected and dried in oven at 50°C to constant weight. The obtained black SLs was then washed with n-hexane to remove oil and dried to constant weight. The crude SLs was again dissolved in 1 ml chromatographic pure acetonitrile and filtered by an organic filtration membrane (0.22 µm). The analytical column of SLs was Venusil MP C18 by HPLC. The mobile phase was acetonitrile and water. Gradient elution (V/V) was used, and the total velocity was 1.0 ml/min. The elution procedure was as follow: 0–15 min, 40–60% acetonitrile;15–30 min, 60–70% acetonitrile; 30–40 min, 70–90% acetonitrile. Acetonitrile was kept at 90% for 5 min. The measured wavelength is 207 nm [1]. The MS analysis of each peak of HPLC was used API 4000 mass spectrometer: the ion source was ESI; Spray voltage was −4500 V. The ion source temperature was 100°C. Atomized gas is 25 psi; the auxiliary gas is 20 psi; Positive ion mode, scanning range: 50–800 amu.

## Results and discussion

### Acacia fermentation with different hydrophobic carbon sources

Different hydrophobic carbon sources affected the yield and composition of SLs [35–39]. Glucose as hydrophilic carbon source, sunflower seed oil, the fried waste oil, cooked tung oil and raw tung oil were used as hydrophobic carbon source for fermentation. It can be seen from the experimental results that when sunflower seed oil was the hydrophobic carbon source, the yield of total SLs and lactone-type SLs was highest from Table 1, and the residual glucose was 1.35 gL^−1^. When fried waste oil was used as the hydrophobic carbon source, the total SLs yield was 39.09 gL^−1^, lactone-type was 10.27 gL^−1^, and the biomass was 8.37 g/L. When cooked tung oil and raw tung oil were used as hydrophobic carbon sources, the yield of total SLs was very low, lactone-type Sophia locust sugar fat was not detected, and the residual glucose was 22.46 and 24.21 gL^−1^, and the biomass was 4.52 and 3.54 g/L. Such results may be caused by the components contained in tung oil. Tung oil was extracted tung tree seeds. They often were divided into raw tung oil and cooked tung oil, and cooked tung oil was from raw tung oil. The main component of tung oil was tung oil acid triglyceride. It includes 18 carbon fatty acids in the conjugate triene acid (α-eleostearic acid and β-eleostearic acid) 79.5%, oleic acid 15.0%, linoleic acid 5.5%. The α-tung acid in tung oil may have some cytotoxicity to *S. bombicola* (CGMCC1576) [40–42].

**Table 1:** Results of SLs fermentation with different hydrophobic carbon sources

Hydrophobic carbon sources	Total SLs (gL^−1^)	Lactone type acacia (gL^−1^)	Glucose residue (gL^−1^)	Biomass (gL^−1^)
Sunflower seed oil	44.52	11.94	1.35	9.21
fried waste oil	39.09	10.27	1.98	8.37
Cooked tung oil	3.23	Not Detected	22.46	4.52
Raw tung oil	2.61	Not Detected	24.21	3.54

Therefore, according the results that there was a difference in the yield of SLs when sunflower seed oil and fried waste oil were the hydrophobic carbon sources. If sunflower seed oil was replaced by fried waste oil as the hydrophobic carbon source, the production cost can be greatly reduced.

### HPLC-MS identification and analysis of SLs with different hydrophobic carbon sources

**Figure 1:**
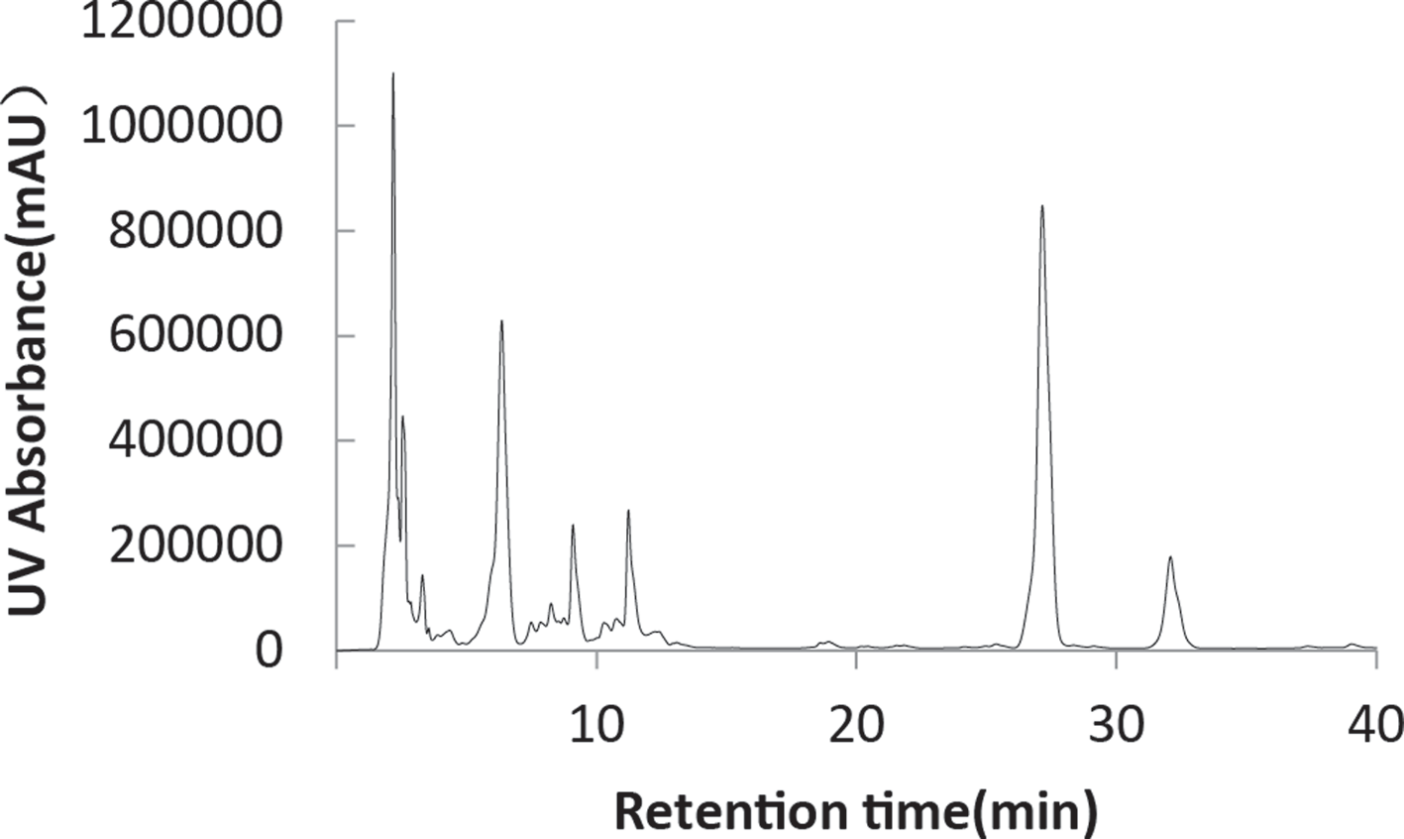
Analysis of SLs by HPLC with sunflower seed oil as the hydrophobic carbon source

**Figure 2:**
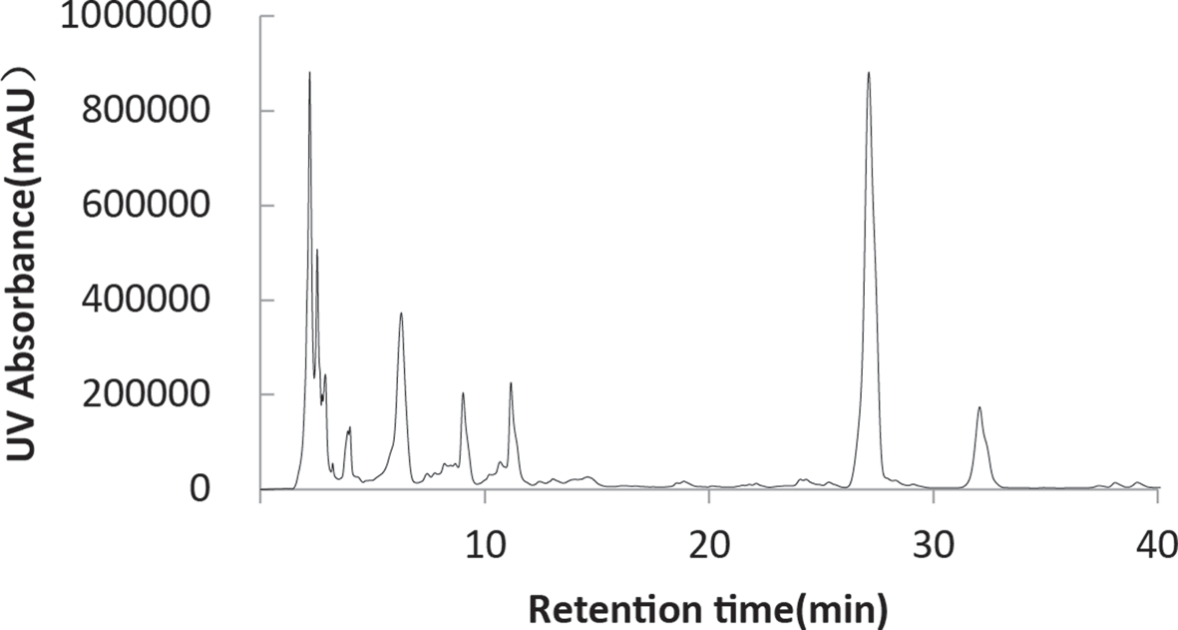
Analysis of SLs by HPLC with fried waste oil as hydrophobic carbon source

According to the above experimental results, sunflower seed oil and fried waste oil were, respectively, used as hydrophobic carbon sources for fermentation by HPLC, and the composition of SLs was analyzed, according Figures 1 and 2. It can be seen that the composition of SLs obtained by two hydrophobic carbon sources fermentation was complex, including more than 20 SLs molecules, and there was little difference in the composition, and both contain the five main components of SLs, the only difference was in the yield.

Five major components of SLs were further analyzed by HPLC-MS. The retention time, molecular ion peak and structure of SLs of each component were shown in Table 2. The 5 kinds of SLs contain was 18 carbon fatty acids, the difference was mainly in the unsaturated fatty acid levels (a double bond or two double bond), the degree of acetylation of SLs (not acetylated, acetyl or diacetyl) and whether there was a lactonization (lactone or acid type).

**Table 2:** Structural identification of several major components of SLs

Sequence number	Retention time (min)	Molecular ion	SLs structure
1	6.36	620	Non-acetylated acidic SL with a C 18:2 fatty acid
2	9.18	662	Mono-acetylated acidic SL with a C18:2 fatty acid
3	11.22	664	Mono-acetylated acidic SL with a C 18:1 fatty acid
4	27.14	686	Di-acetylated acidic SL with a C 18:2 fatty acid
5	30.07	688	Di-acetylated acidic SL with a C18:1 fatty acid

The peak 1 was the side chain as linoleic acid of acetylated acid type of SLs, single peak 2 was the side chain as linoleic acid acetylated acid type, peak 3 was the side chain as the single acetylated acid oleic acid, peak 4 was the side chain as linoleic acid lactone type of SLs, and peak 5 was the side chain as diacetyl of oleic acid lactone type of SLs.

### Optimization of fermentation medium

Using the fried waste oil as the hydrophobic carbon source, single factor optimal fermentation conditions was designed on the basis of medium composition glucose, fried waste oil and yeast powder. The results were shown in Table 3.

**Table 3:** Orthogonal test results of fermentation medium

Test no.	Glucose/%A	Fried waste oil/%B	Yeast powder/%C	Total SLs (gL^−1^)
1	1(6)	1(6)	1(0.2)	26.53
2	1	2(8)	2(0.3)	49.12
3	1	3(10)	3(0.4)	24.94
4	2(8)	1	2	76.14
5	2	2	3	66.73
6	2	3	1	40.45
7	3(10)	1	3	36.38
8	3	2	1	18.96
9	3	3	2	32.70
*K*_1_	100.59	139.05	85.94	
*K*_2_	183.32	134.81	157.96	
*K*_3_	88.04	98.09	128.05	
*R*	82.73	40.96	72.02	

According to Table 3, the optimal combination of fermentation medium with fried waste oil as hydrophobic carbon source was A2B1C2, which was glucose 8%, fried waste oil 6%, and yeast powder 0.3%. The results of range *R* value analysis showed that the effect order of fermentation medium on the yield of SLs was A>C>B, that mean, the affecting order of the yield of SLs: glucose > yeast powder > fried waste oil.

According to Table 4, the influence order of fermentation medium on the yield of SLs was consistent with the orthogonal test results. From the ANOVA table (Table 4), it can be concluded that the significance level of glucose was all less than 0.05 with extremely significant differences.

**Table 4:** Analysis of the effects of different glucose, fried waste oil and yeast powder levels on the yield of SLs

Source of variation	Sum of squares	Degree of freedom	Mean square	*F*-value	Significance level
Model	2997.648*	6	499.608	128.517	0.008
Glucose	1786.670	2	893.335	229.798	0.004
fried waste oil	338.229	2	169.114	43.502	0.022
Yeast Powder	872.749	2	436.374	112.251	0.009
Error	7.775	2	3.887		
total	18377.290	9			
Corrected Total	3005.423	8			

* *R*^2^ = 0.997.

### Test results of the SLs yield using fried waste oil by four-strand parallel fermentation tanks

**Figure 3:**
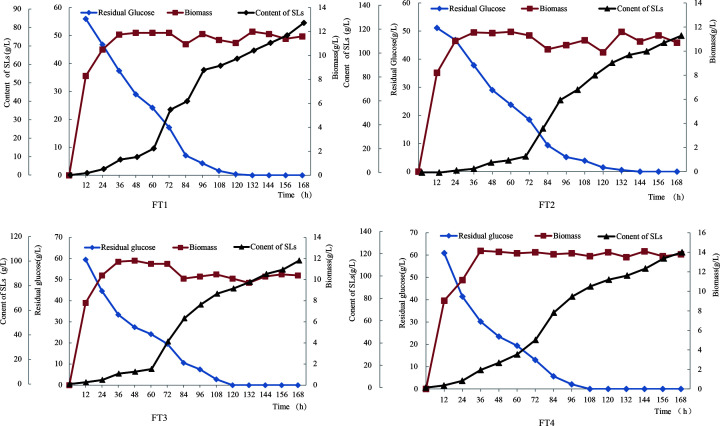
Production of SLs by four parallel fermentation tanks

Four different kinds of fermentation were be carried out at the same time by using a parallel quad fermentation tank. The production capacity of pseudocandida *S. bombicola* (CGMCC1576) with different amount of waste oil was investigated. The results of the design test were shown in Figure 3.

**Figure 4:**
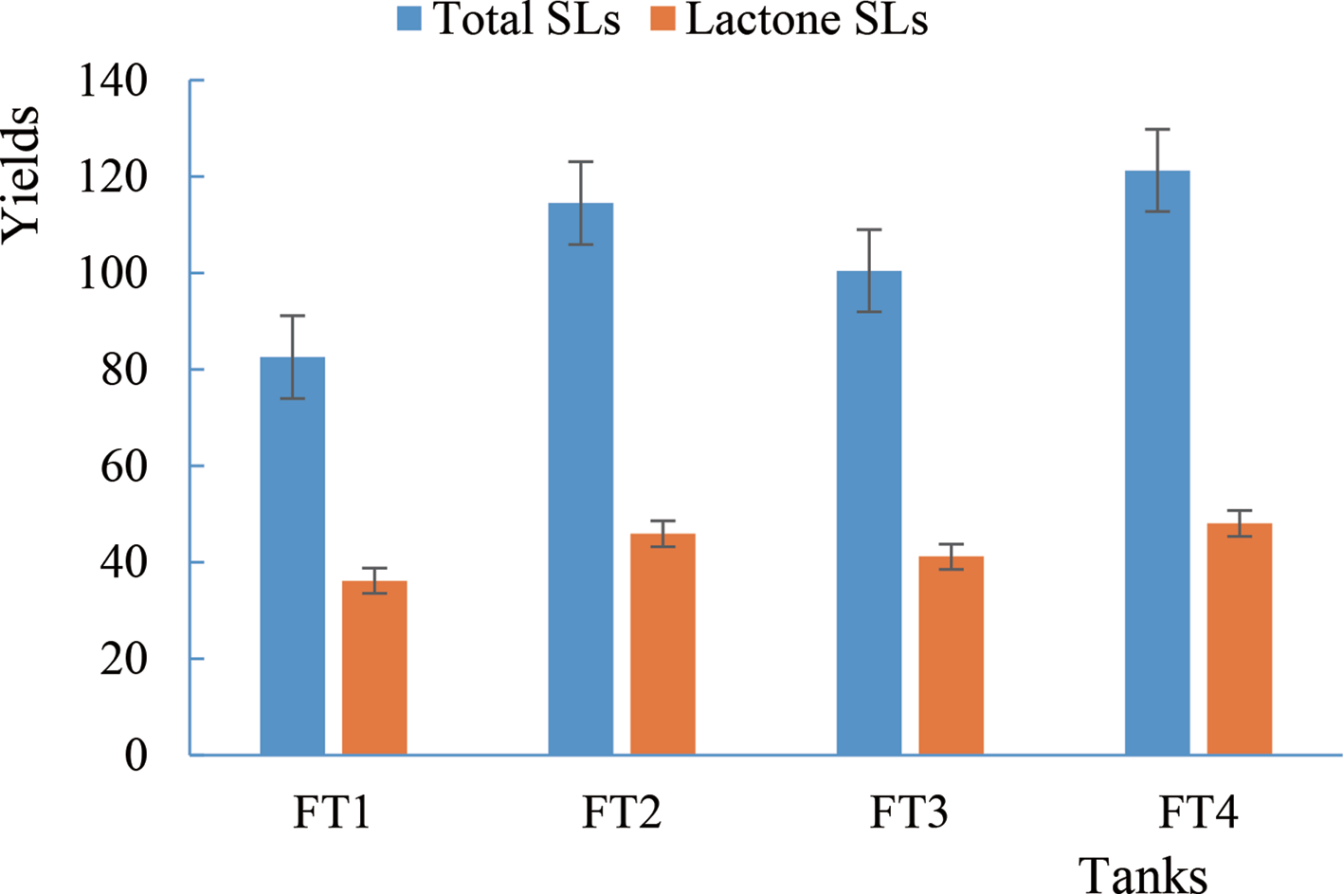
Results of total SLs and lactone SLs produced by four parallel fermenting tanks

From the Figure 3, the biomass of four different fermentation tanks increased rapidly within 48h after inoculation, and remained unchanged basically from 48h to the end of fermentation. During this period, the biomass of tank 4 was larger than that of the other three, with a maximum value of 14.2 gL^−1^. The glucose in tank 1, 2 and 3 was basically used up after 120 h, and that in ferment tank 4 was used up in advance of the others when 108h. As can be seen from Figure 4, all the four fermentation tanks produced SLs, and the yield was significantly increased after 72 h of fermentation in fermentation tank 2, tank 3 and tank 4, while no such phenomenon was in tank 1.

From the Figure 4, the yield of SLs in the fermentation tank 1 was 82.54 gL^−1^, that of tank 4 was 121.28 gL^−1^, and 31.94% higher than tank 1, it showed that flowing with supplementary food fermentation production of SLs was higher score of batch fermentation. The main cause of this result was fed-batch the hydrophobic carbon source limitation of dissolved oxygen mass transfer rate, and improved the supply efficiency of dissolved oxygen in fermentation reaction system. Thus, effectively improved the hydrophobic carbon conversion rate. The amount of biomass and residual glucose in fermentation tank 4 also fully proved this conclusion from Figure 4.

From the above analysis, it can be concluded that the best and most cost-effective method to produce SLs by adding different amount of fried waste oil with *S. bombicola* (CGMCC1576) was to add 3% of fried waste oil into the initial medium, and then add 3% of fried waste oil after 72 h fermentation.

## Conclusion

*S. bombicola* (CGMCC1576) could use sunflower seed oil and Fried waste oil as hydrophobic carbon sources to produce SLs, and the total yield was 44.52 g/L and 39.09 g/L respectively. From the data analysis, there was no significant difference in the total production of SLs when fried waste oil and sunflower seed oil were used as hydrophobic carbon source, and the main composition and structure of SLs produced by using fried waste oil as hydrophobic carbon source were similar to those produced by using sunflower oil. The cost of fermentation medium in fried waste oil for the hydrophobic carbon source was lower than in sunflower seed oil, it showed that application of fried waste oil for the hydrophobic carbon source can not only effectively reduce SLs but also realize resource and free-pollution disposal of fried waste oil, so it has good economic benefit and environment effect.

*S. bombicola* (CGMCC1576) could not use cooked and raw tung oil as hydrophobic carbon sources to produce SLs, which might be caused by the composition in tung oil. Tung oil was extracted from the seeds of the oil tree, divided into raw tung oil and cooked tung oil. α-Tung acid in tung oil may have certain cytotoxicity to *S. bombicola* (CGMCC1576).

The optimal combination of fermentation medium with fried waste oil as hydrophilic carbon source was 8% glucose, 6% fried waste oil and 0.3% yeast powder. The order of the effect of fermentation medium on the yield of SLs was the amount of glucose > yeast powder > fried waste oil.

The yield of SLs was higher than batch fermentation when flowing adding supplementary food application with four parallel FT using fried waste oil as hydrophobic carbon source. The combination of the largest yield and most cost-saving for initial culture medium was adding 3% of the fried waste oil, flowing add 3% the fried waste oil again after 72 h, and total yield of SLs was 121.28 g/L, lactone type of SLs was 48.07 g/L.

## Data Availability

All data are available in the manuscript text.

## Abbreviations

FT: fermentation tank
HPLC-MS/MS: high-performance liquid chromatography/high resolution mass spectrometry
SL: sophorolipid
